# Chronic Pain as a Hypothetical Construct: A Practical and Philosophical Consideration

**DOI:** 10.3389/fpsyg.2017.00664

**Published:** 2017-04-27

**Authors:** Daniel M. Doleys

**Affiliations:** Doleys Clinic/Pain and Rehabilitation Institute, BirminghamAL, USA

**Keywords:** chronic pain, alternative conceptualization, hypothetical construct, intervening variable, emergent phenomenon

## Abstract

Pain has been defined by the International Association for the Study of Pain (IASP) as “an unpleasant sensory and emotional experience associated with actual or potential tissue damage, or described in terms of such damage.” Chronic pain is usually described as pain that has persisted for 3–6 months and/or beyond the expected time of healing. The numerical pain rating (NPR) is the customary metric and often considered as a proxy for the subjective experience of chronic pain. This definition of pain (chronic) has been of significant heuristic value. However, the definition and the models it has spawned tend to encourage the interpretation of pain as a measurable entity and implies that the patient’s experience of pain can be fully comprehended by someone other than the person in pain. Several major models of pain have been scrutinized and found to propagate the notion of pain as a ‘thing’ and fall prey to biomedical reductionism and Cartesian (mind-body) dualism. Furthermore, the NPR does not appear to capture the complexity of chronic pain and correlates poorly with other clinically meaningful outcomes. It, and other aspects of the current notion of chronic pain, appear to be an extension of our reliance on the philosophical principles of reductionism and materialism. These and other shortcomings identified in the IASP definition have resulted in an increased interest in a reexamination and possible updating of our view of pain (chronic) and its definition. The present paper describes an alternative view of pain, in particular chronic pain. It argues that chronic pain should be understood as a separate phenomenon from, rather than an extension of, acute pain and interpreted as a hypothetical construct (HC). HCs are contrasted to intervening variables (IV) and the use of HCs in science is illustrated. The acceptance of the principles of nonlinearity and emergence are seen as important characteristics. The practical implications and barriers of this philosophical shift for assessment, treatment, and education are explored. The patient’s narrative is presented as a potential source of important phenomenological data relating to their ‘experience’ of pain. It is further proposed that educational and academic endeavors incorporate a discussion of the process of chronification and the role of complexity theory.

“My realism about the subjective domain in all its forms implies a belief in the existence of facts beyondthe reach of human concepts”

(Nagel T. What is it like to be a bat? Philosophical Review, 1974, 33, p. 437)

## Background

The International Association for the Study of Pain (IASP) defines pain as a “sensory and emotional experience…” (Merskey and Bogduk, 1994). Chronic pain has customarily been understood as pain that has persisted for 3–6 months and/or beyond the expected time of healing (International Association for the Study of Pain, 1986). Bonica and Livingston introduced the multidisciplinary philosophy and approach to chronic pain in the 1950s (Bonica, 1974). Since then, there has been a proliferation of organizations/societies, journals/books, and clinicians/clinics devoted to the assessment and treatment of chronic pain. Technological and pharmaceutical companies have vigorously pursued the development of procedures, hardware, and pharmaceutical agents designed to address the presumed physiological, anatomical, and pharmacological basis of chronic pain. A variety of behavioral/psychological models, assessments, and treatments have emerged over the decades designed to equip and empower the patient with self-directed therapies intended to alter the subjective level and overall experience of chronic pain.

Despite these efforts, The Institute of Medicine [IOM] (2011) report and a review by Turk et al. (2011) give little indication that the current therapies yield results which are clinically meaningful to the patient and economically meaningful to society. Furthermore, epidemiological data suggests that the incidence and prevalence of chronic pain is increasing across time (Institute of Medicine [IOM], 2011). This trend is consistent among various age groups. For example, one report noted a nine-fold increase in hospital admission for children and adolescents with some type of chronic pain diagnosis (Thomas et al., 2013). Whether this represents increased awareness, detection, and/or reporting is unclear.

The basic science of pain in general has witnessed remarkable changes over the decades. Research into the neuroanatomy and neurophysiology of the sensory system has greatly expanded our understanding of the complexities of the mechanisms of transduction and transmission (Woolf, 2007; Basbaum et al., 2009; Stokes et al., 2013). Advancements in neuroimaging techniques have led to the uncovering of a dynamic interplay among various cortical regions involved in the processing of incoming information (Apkarian et al., 2005, 2009; Tracey and Mantyh, 2007; Nash et al., 2013) and the nature of neuroplasticity (Martucci et al., 2014). Davis et al. (2015) described pain as, in part, being “… binary and complexly encoded” and encouraged pain researchers to find the “…neural code of the pain switch” (p. 2165). However, the anatomy and physiology of pain does not explain what it is like to be IN pain. The overall experience of chronic pain is not adequately represented by elaborating the electrophysiological activity in the nociceptive systems. The psychological, functional, and dynamic aspects can easily be overlooked or minimized.

The 2008 Pain Terminology group (Loeser and Treede, 2008) emphasized that pain and nociception should not be confused, as one can occur without the other. They also introduced the term ‘nociceptive stimulus’ as “…an event transduced and encoded by nociceptors” and distinct from other events, which “…although causing tissue damage, are not detected by any sensory receptor and therefore do not cause pain” (p. 475). The discussion was heavily imbued with references to sensory physiology and psychophysical relationships. Although psychophysical laws express regularities between a stimulus (i) and percepts, they do not account for why the percept has the qualitative character it does or why it has any qualitative character at all. Furthermore, the development, mechanism(s), and role of the non-conscious processing of sensory, affective, and motivational information and the manner in which this becomes a conscious experience, though critical to understanding pain, has received relatively little attention.

Given the subjective nature of pain, researchers and clinicians seem compelled to rely upon the patient (at least those possessing sufficient communicative abilities) to designate their pain via a numerical pain rating (NPR). This approach maintains the focus on ‘the pain,’ as defined by the subjective rating, and the presumption that reducing the NPR will normalize the associated components including one’s quality-of-life. Despite the lack of correlation between statistically significant changes in the subjective pain rating and improvements in functioning (McCracken et al., 2002; Jensen et al., 2004; Shah et al., 2015) as well as patient satisfaction with treatment (Comley and DeMeyer, 2001), there appears to be a rigid adherence to changes in NPR scores as the primary indicator of therapeutic efficacy and effectiveness (Farrar et al., 2001; Ballantyne and Sullivan, 2015; Sullivan and Ballantyne, 2016). Admittedly, the use of multidimensional outcomes is encouraged (Gewandter et al., 2014), but they are frequently considered secondary to a reduction in the pain intensity rating.

The relevance and meaning of the time-honored NPR has recently been called into question (Farrar et al., 2001; Ballantyne and Sullivan, 2015; Sullivan and Ballantyne, 2016). Backonja and Farrar (2015) noted “…the complexity of the human pain experience reminds us that we neither have a clearly articulated nor widely accepted statement about what the pain intensity ratings represent” (p. 1247). It should not be surprising that chronic pain, as a subjective experience, would be difficult to quantify.

The ease with which we can manipulate numbers provides a false sense of security about our level of knowledge and understanding as it relates to the experience the number is purported to represent. Any attempt to understand another’s pain, chronic or otherwise, based upon a numerical score appears to encounter the same difficulties. Nagel (1974) outlined is his article summarizing the problems of imagining what it is like to be a bat and Quintner et al.’s (2008) notion of pain as an aporia. Both instances reflect a state of impasse in the current level of knowledge and understanding.

The persistent and apparent unyielding reverence given the NPR seems to be undergirded by a continued emphasis on the philosophical principles of materialism and reductionism. The NPR is often interpreted as a very fundamental and objective representation of the patient’s experience. This is reflected by the NPR being heralded as the ‘fifth vital sign’ (Campbell, 2016) and a required, if not a primary, research outcome measure. Ironically, its pervasive use in the clinical setting has even been linked to the remarkable increase in opioid prescribing (Farrar et al., 2001; Sullivan and Ballantyne, 2016).

Given the current state of knowledge, it would seem somewhat parochial to consider pain as existing on a continuum from acute pain–chronic pain. That is, chronic pain is pain which has lasted 3–6 months and/or persisted beyond the expected time of healing. The appreciation of pain as a disease process and the dynamic nature of neuroplasticity supports the consideration of chronic pain as a separate but related phenomenon (state) from acute pain. Furthermore, the relative lack of a positive correlation between the degree of physical damage, the NPR, and other psychological/functional aspects of chronic pain, suggests these relationships are not linear. Nonlinearity and other aspects of dynamical systems theory have been applied previously in the analysis of human behavior in general (Barton, 1994) and pain in particular (Young and Chapman, 2006, 2007; Chapman et al., 2008). The use of elements of systems theory has been found to describe processes which are not effectively described using a more linear approach.

The process of chronification (Institute of Medicine [IOM], 2011; Granan, 2016) may represent the mechanism of transition from one clinical state to another: acute to chronic. The ontological distinctiveness of neuropathic, inflammatory, and cancer-related pain is becoming more apparent (Honore et al., 2000; Hunt and Mantyh, 2001; Baliki et al., 2011). However, emphasizing terms such as nociceptive, neuropathic, and inflammatory maintains the focus upon the peripheral activity presumed to be stimulating the nociceptive system. This emphasis overlooks the transitioning from a sensory to affective system which composes a significant aspect of the processes of neuroplasticity and chronification (Hashmi et al., 2013). These observations indicate the need for caution when attempting to generalize research findings from the acute to the chronic pain state and from one category of pain to another. The complexity and uniqueness of the chronic pain experience also raises the question as to degree one can generalize from the animal to the human, the lab to the clinic, and the healthy volunteer to the patient.

In their review of contemporary biopsychosocial models of pain, Quintner et al. (2008) noted a tendency toward biological reductionism and the continued absence of an approach that captures the “…lived experience of pain as an emergent and unpredictable phenomenon” (p. 825). Although the principles of reductionism and materialism have guided scientific research for centuries, they should not define the boundaries of our thinking about chronic pain. The adherence to a purely neurophysiological approach may have a certain appeal from an aesthetic perspective, but may not capture the totality of the pain ‘experience’ (Thacker and Moseley, 2012). This approach may unduly emphasize the pain and not the patient with pain (Sternbach, 1974). Consideration should be given to the possible contributions of other areas such as systems theory and quantum theory to our understanding of chronic pain (Doleys, 2014).

The above observations argue for a reconsideration of our perspective of chronic pain. This brief commentary is designed to provide a philosophical rationale for a description of a different paradigm within which to view chronic pain. The intent is to stimulate thought regarding that which we are treating rather than merely expanding the ways in which we treat it.

## Hypothetical Constructs and Intervening Variables

From an epistemological perspective, our understanding of the nature of pain continues to evolve. In the 20th century, the progenitor for this evolving perspective has been the gate control theory (Melzack and Wall, 1965) and the subsequent introduction of the neuromatrix theory (Melzack, 2001). Pain has been described as a homeostatic dysfunction (Loeser and Melzack, 1999), a homeostatic emotion (Craig, 2003), a disease process (Siddall and Cousins, 2004), and a destructive disease process (Ballantyne, 2006). Carr (1993) noted pain to be “a dynamic process that actions at multiple sites ranging from the peripheral nociceptor to the genome of cells within the central nervous system to the patient’s psychosocial milieu.” Most recently, Williams and Craig (2016) suggested amending the definition of pain to include the phases “…distressing experience” and “…cognitive and social components.” In fact, Melzack (1989) declared that one does not have to possess a physical body to experience pain, e.g., phantom pain. Therefore, the current understanding of pain may also benefit from a reexamination.

In their discussion of philosophical and methodological issues relating to scientific research and discourse, MacCorquodale and Meehl (1948) and Cronbach and Meehl (1955) drew a distinction between intervening variables (IV) and hypothetical constructs (HC). An IV is one which has been systematically defined in terms of its antecedents and is dependent upon these for its meaning. IVs represent a convenient or short-hand way of abstracting an empirical relationship and have no meaning apart from that relationship.

That is to say, IVs have no physical or psychological reality. They have no factual content beyond the empirical functions they serve to summarize. An IV is usually introduced as a means of simplifying the written expression of the empirical relation to which it is attached. However, a HC has no single referent. The construct consists of groups of functionally related behaviors, attitudes, processes, and experiences.

Hypothetical constructs “…involve terms that are not wholly reducible to empirical terms; they refer to processes or entities that are not directly observed” (p. 104) and the validity of the empirical law is not a sufficient condition to establish the truth of the construct. HCs are described as containing ‘surplus meaning’ and cannot be explained, nor do they equate to, the sum of the variables contained in an empirical relationship. A HC is a conjectured entity, process, or event that although not directly observed is assumed to explain an observable phenomenon. Not only do HCs contain a supposition of entities or process not among the observed, they “…have a cognitive, factual reference in addition to the empirical data, which constitutes their support… and their actual existence should be comparable with general knowledge” (p. 107).

Because these inferred variables are not wholly contained in their antecedents, it follows that the meaning, or ontological content, of the inferred variable is not reducible to that of its antecedents. MacCorquodale and Meehl (1948) used concepts from physics as illustrations. The resistance of a wire is considered to be an IV as it merely specifies the amperage of current that will be carried by the wire for any given voltage. However, an electron represents a HC in that it is assumed to be an entity, even though it has not been observed directly. Other examples of HC include genes and evolution from biology, black holes and dark matter from astrophysics, and personality and mood in the area of psychology. Quarks and gluons provide additional examples of HCs. Although they are elementary particles of nuclear material and basic to quantum theory, they are described as “unseen realities” (Polkinghorne, 2007). Likewise, Sternbach (1974) noted that pain had no existence of its own and should be considered as an abstract construct.

For chronic pain to be viewed as an IV it would have to conform to a relationship-dependent-upon-antecedent-conditions and some sense of proportionality. It would violate the tenets of IVs to entertain the notion of chronic pain without sensory input, such as that found in pain associated with social rejection, congenitally absent limb, empathy, hallucinations, and depression. Furthermore, the notion of chronic pain as an IV would have to include all interoceptive and exteroceptive input as objective and quantifiable. Even so, it would implicate chronic pain as equivalent to the sum of its parts and that we know what all the parts are. This idea appears contrary to the ‘subjectiveness’ of the experience of chronic pain. It suggests that once we clarify the components and their mathematical relationship that we will fully comprehend chronic pain. This leaves little or no room for any uniquely private and intra-personal aspect to the experience of chronic pain. MacCorquodale and Meehl’s (1948) notion of ‘surplus’ meaning recognizes the elements of subjectivity and nonlinearity. Therefore, it is proposed that chronic pain, and perhaps pain in general, can most accurately be described as a HC.

As a HC, chronic pain would be understood as an emergent property of the ‘system’ and one that is more than the sum of the parts. Rather than chronic pain being merely a collection of sensory, affective, and evaluative variables, it would be conceptualized as the time-dependent outcome of the dynamic interaction of these variables. The complexity of the system, animal vs. human, and the dynamic relationship among the component parts would impose boundaries on the preciseness of our comprehension and thus our predictions. In his discussion of the nature of scientific theory, Nagel (1961) addressed this point when he asserted that it is possible, indeed plausible, to lack the necessary capacity to fully comprehend a phenomenon despite having the facts at hand.

## An Alternative View

The notion of pain (chronic) as a HC seems in keeping with Wall’s (1979) description of pain as more akin to a ‘need state’ like hunger and thirst than a sensory input signal such as vision or hearing. He also emphasized “…a weak connection to the injury but a strong connection to a body state” (p. 253). Chronic pain as a HC would consist of a constellation of nociceptive, behavioral, functional, mood, as well as other variables, and not merely an extension of acute pain. The variable subsuming a position of supremacy, if there was one, in understanding a given individual is likely to change with their circumstances. That is, for any particular patient their mood may be much more relevant to their experience of chronic pain than the degree of nociceptive input. No single variable or factor would be accorded a position of permanent superiority over the others. From a treatment perspective, this becomes somewhat complex and complicated as the target(s) of intervention may change from time-to-time. The need for ongoing monitoring of the patient and updating of the treatment plan is not unlike that of any chronic illness or disease. However, daunting this may sound, it does seem to accurately reflect clinical reality as outlined by national guidelines (National Guideline Clearinghouse [NGC], 2005; UMHS Chronic Pain Management Guideline, 2016).

When assessing chronic pain as a HC, focusing on the nociceptive component would take on various levels of importance depending upon the outcome of the evaluation. In any case, addressing the nociceptive aspects of the pain processing system through such treatments as pharmacological agents and interventional therapies would be carried out and deemed effective only if they are associated with improving the overall condition of the patient. To the extent that the patient’s quality-of-life is significantly compromised and relatively unaffected by these therapies, they would be considered ineffective or contraindicated. Additionally, the use of such therapies in isolation of other approaches designed to address various components of chronic pain would be deemed inadequate.

The understanding of chronic pain as HC places the emphasis upon the identification of its constituent parts, which then become the focus of treatment rather than the NPR. Therefore, it recognizes the dynamic interaction among these parts and the possibility of the need for continuous intervention at some level (i.e., self-directed); not unlike the need for life-long dietary control and exercise to effectively control diabetes. Viewing chronic pain as a HC promotes it as an emergent property of constituent parts interacting in a complex and dynamic fashion, influenced by factors internal and external to the organism. Interpreted in this way, chronic pain and its management is much more consistent with growing recognition of chronic pain as a dynamic disease process (Tracey and Bushnell, 2009). It also repels any attempts at the application of Cartesian dualism wherein a distinction or deference is given to the source of the input, i.e., body or mind. Furthermore, although attempts to quantify the various components of chronic pain for purposes of research may take place, objectivity (materialism) is a not a requirement for inclusion.

## Implications

The assessment of chronic pain from the perspective of a HC should be expanded to include quantitative and qualitative data. The use a narrative analysis (Carr et al., 2005; Englander, 2012; Morris, 2012) may provide important experiential and phenomenological information, seemingly less objective but potentially more relevant and representative of the experience of pain. Outcome assessment should incorporate the patient’s perception of their ‘overall’ change rather than forcing a selection from predetermined choices characteristic of many questionnaires/tests and the NPR scale (Williams et al., 2000).

Understanding chronic pain as a HC has implications for treatment but would face some barriers. The general overreliance upon physiologically based treatments by various types of clinicians and the enthusiastic acceptance of such treatments by patients desperate for a cure will require a significant philosophical shift and reeducation. In part, this can be approached by presenting chronic pain as a different phenomenon rather than an extension of acute pain. The emphasis would be placed on increasing patient participation and responsibility. Many of the more physiologically oriented treatments inadvertently encourage the patient to become overly dependent upon the clinician and the risk of the clinician becoming co-dependent with the patient. Within the concept of chronic pain as a HC, smoking reduction, weight control, nutrition, functional restoration, and stress management might be considered essential parts of the overall treatment to the extent they are deemed to be addressing factors relevant to the development/maintenance of the chronic pain condition. The absence of patient participation in these activities or a lack of improvement in the context of more physiologic interventions would result in a reevaluation of the therapeutic algorithm.

**FIGURE 1:**
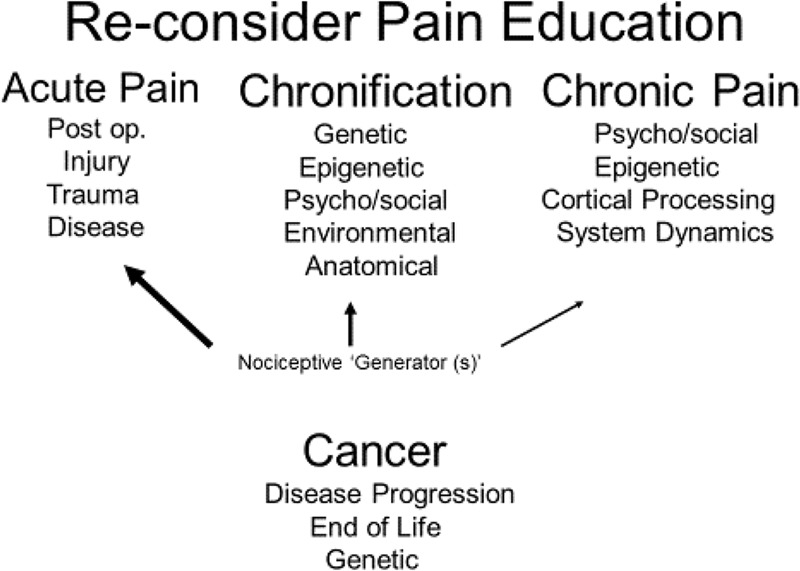
**Illustrates the recommended approach to pain education based on chronic pain as a hypothetical construct (HC).** Rather then a continuum, acute and chronic pain should be approached as different states. Chronification is the mechanism by which acute pain emerges into a chronic disease state. Because of its nature, cancer-related pain would incorporate aspects of all three states. The terms under each heading reflect possible content areas. The diminishing size of the arrows extending from Nociceptive Generators indicates that the role of sensory input is likely to become less influential in accounting for the patient’s chronic pain experience compared to acute pain.

The educational approach to chronic pain would also require some reconsideration. Rather than producing materials which portray a somewhat linear progression from peripheral nociception to acute pain to chronic pain, the areas of acute pain, chronification, and chronic pain should be presented as distinct but related entities (**Figure 1**). Pain related to cancer would encompass all three areas. Assessment/treatment strategies and outcomes which have found meaning in the acute setting may not translate to the chronic pain state. The complexities and dynamics of chronic pain, in light of the fact that it may exist for the life of the patient, may require something akin to a life-course approach (Ben-Shlom and Kuh, 2002) which is consistent with the growing recognition of chronic pain as a progressive disease process (state).

## Summary

Attempts to understand pain within the IASP definition has generated numerous theories and models. Most have focused on enumerating elements of the neurophysiological and behavior/psychological aspects of pain. Despite, or perhaps because of, the acknowledged subjectivity of pain, quantification of these elements and their relationship has been a prime objective. The patient’s subjective rating has often assumed a position of prominence in describing the patient’s experience and supporting the existence of the supposition of pain as an actual entity.

Although rarely discussed in the scientific literature, the absence of proportionality has stimulated an interest in alternative approaches. The use of dynamical and complexity theory with its acceptance of nonlinearity and emergence is one such approach. Within this framework pain, especially chronic pain by virtue of its complex nature, can most easily be understood as a HC. This is in keeping with the inconsistent correlations among independent and dependent variables as noted above, and the contextual sensitivity of chronic pain. As a HC chronic pain is recognized as more than the sum of its parts, complex and dynamic in its nature, and as an emergent phenomenon.

This conceptualization has implications for assessment, treatment, and education. The assessment should include an analysis of the patient’s own narrative rather than relying solely on standardized measures and the NPR. Re-assessment of the therapeutic algorithm will be needed to accommodate changes in the patient’s circumstances, which in turn can influence the therapeutic target and technique. Self-directed assessment and treatments would be emphasized as treatment is expected to be indefinite. The academic approach should be modified to included chronic pain as a product of chronification and distinct from acute pain. The philosophical issues involved in understanding pain should also be discussed.

The notion of chronic pain as a HC is theoretically testable. Comparing the results of allowing patients greater latitude in depicting the nature of their pain experience, selecting desired treatment goals, and a preferred course of therapy to the more classical predetermined protocol would be one mechanism for testing the validity of the approach presented herein.

Chronic pain is uniformly characterized as a multifactorial phenomenon. Most would agree that it incorporates not only sensory but also functional and psychological components. The interrelationship among these variables is influenced by a multitude of intra- and inter- personal factors/processes, including chronification and neuroplasticity and will manifest an element of unpredictability. The continued search for linearity seems inconsistent with the clinical narrative, and may be misguided. If, as it appears, the number of factors that contribute to the experience of chronic pain, the relative contribution of any particular factor, and the manner in which these factors interrelate represent aspects of a complex and dynamic system, chronic pain would be more accurately understood as a HC.

## Author Contributions

The author confirms being the sole contributor of this work and approved it for publication.

## Conflict of Interest Statement

The author declares that the research was conducted in the absence of any commercial or financial relationships that could be construed as a potential conflict of interest.
